# Cichlid fishes are promising underutilized models to investigate helminth-host-microbiome interactions

**DOI:** 10.3389/fimmu.2025.1527184

**Published:** 2025-02-13

**Authors:** Maarten P. M. Vanhove, Stephan Koblmüller, Jorge M. O. Fernandes, Christoph Hahn, Michelle Plusquin, Nikol Kmentová

**Affiliations:** ^1^ Research Group Zoology: Biodiversity and Toxicology, Centre for Environmental Sciences, Hasselt University, Diepenbeek, Belgium; ^2^ International Union for Conservation of Nature (IUCN) Species Survival Commission (SSC) Parasite Specialist Group, Diepenbeek, Belgium; ^3^ Institute of Biology, University of Graz, Graz, Austria; ^4^ Renewable Marine Resources Department, Institut de Ciències del Mar, Spanish National Research Council, Barcelona, Spain; ^5^ Research Group Environmental Biology, Center for Environmental Sciences, Hasselt University, Diepenbeek, Belgium; ^6^ Freshwater Biology, Operational Directorate Natural Environment, Royal Belgian Institute of Natural Sciences, Brussels, Belgium

**Keywords:** Cichlidae, immunomodulation, Lake Tanganyika, microbial diversity, symbiome, old friends hypothesis, parasite, worm

## Abstract

The “Old Friends Hypothesis” suggests insufficient exposure to symbionts hinders immune development, contributing to increased immune-related diseases in the Global North. The microbiome is often the focus; helminths, potentially also offering health benefits, lack attention. Infection and effect of helminths are influenced and perhaps determined by micro-organisms. Mechanisms behind parasite-microbiome interactions are poorly understood, despite implications on host health. These interactions are typically studied for single helminth species in laboratory animal models, overlooking helminth diversity. Reviewing research on relationships between helminth and microbial diversity yielded 27 publications; most focused on human or other mammalian hosts, relying on natural exposure rather than experimental helminth inoculation. Only about half investigated host health outcomes. Remaining knowledge gaps warrant considering additional candidate model systems. Given the high helminthiasis burden and species diversity of helminths, we propose seeking models in the Global South, where a considerable proportion of research on diversity aspects of helminth-microbiome interactions took place. Low availability of genomic resources for helminths in the Global South, however, necessitates more integrative helminthological research efforts. Given substantial similarities in immune systems, several fishes are models for human health/disease. More effort could be done to establish this for cichlids, whose representatives in the African Great Lakes provide a well-delineated, closed natural system relevant to human health in view of fish-borne zoonoses and other water-borne parasites. A good baseline exists for these cichlids’ genomics, parasitology, and microbiology. We suggest exploring African Great Lake cichlids as model hosts for interactions between microbial diversity, helminth diversity, and host health.

## 1 Introduction

From birth onwards, humans are colonized by microorganisms including fungi, protozoans and viruses (1). In addition, humans have coexisted with helminths for most of our existence (2). Yet, since the industrial era, countries in the Global North have established a reduction in prevalence of helminths and other parasites (3, 4). Additionally, studies have observed an association between industrial lifestyles and reduced diversity in the gut microbiome (5). Allergic disorders and inflammatory conditions increased throughout the 20^th^ century in the Global North (6, 7). From the late 19^th^ century, hay fever was increasingly reported in wealthy urban populations, while farming communities remained less affected (8). As living conditions have changed, these trends indicate a disturbance in immune regulation, leading to a disbalanced response to harmless allergens or infections.

The “Old Friends Hypothesis” provides an evolutionary view on the rise of immune-related disorders like allergies, asthma, and autoimmune diseases, including a focus on early life (9). It describes that the absence or reduced exposure to specific microorganisms or helminths in early life, which co-evolved with humans as hunter-gatherer omnivores, disrupts normal immune system development. This may lead to a disturbance of immunoregulation due to insufficient exposure to microorganisms or helminths that drive the expansion of components such as regulatory T cells (10). The hypothesis suggests that exposures to specific organisms are critical for properly training the immune system to differentiate between harmful and benign antigens, and preventing excessive immune responses. For example, lipopolysaccharide, a component of the outer membrane of gram-negative bacteria, may protect against allergic responses by inducing the ubiquitin-modifying enzyme A20 in lung epithelial cells (11). Also, helminths seem to fit the narrative of the “Old Friends Hypothesis”. For instance, helminth infections may also confer protective benefits against autoimmune diseases, allergies, and inflammatory disorders by promoting immune tolerance through immune system modulation and microbiome alterations (12–14). In laboratory animal models, helminth infections have been linked to suppression of pro-inflammatory gut bacteria, leading to protection against Crohn’s disease in genetically susceptible mice (15). Similarly, in humans, helminth infections have been shown to increase microbial diversity and alter antibody levels, although the full extent of these effects is still not well understood (16). Infection with pinworms (*Enterobius vermicularis*) was shown to be associated with a protective effect on the development of clinical malaria in Tanzanian children, while infection with hookworm exacerbates the severity of malaria (17).

Several studies indicate that helminth infections may have a substantial effect and change the diversity and composition of gut microbiota (18–21). These shifts in the microbiome could affect the risk for various diseases, such as asthma, viral infections, and metabolic disorders (22, 23). Furthermore, the microbiome plays a role in the establishment of certain helminth infections and can influence their progression (24). Specific bacterial taxa have been found to affect an individual’s resistance or susceptibility to helminths (25), though the precise mechanisms underlying these helminth-microbiota interactions remain largely unclear. Despite the promising findings that infection with helminth parasites alleviates symptoms of various diseases, our understanding of the mechanisms underlying the interaction between parasites and the microbiome remains limited.

## 2 The study of microbiome-parasite interactions needs more attention for parasite and host diversity, which relies on natural exposure

Despite their interconnected roles in host health, microbes and parasites have traditionally been studied in isolation, overlooking their collective contribution as part of a “symbiome”. We regard the term “symbiont” (and collectively “symbiome”) as referring to any host-associated organisms (including viruses), which can have negative, neutral, or positive interactions with the host (26).

According to the holobiont concept (27, 28), the symbionts and the host act as a unique biological entity termed “holobiont”, a unit of selection in evolution (29). Consequently, the “hologenome” is the collective of genomes of host and symbiome, with interactions between hologenome and environment determining phenotype and being pivotal to adaptation and evolution (30, 31). Symbiome genomes can change rapidly under environmental stress and therefore buy the host precious time to adapt and survive (32). Hologenomic approaches might be applied as part of the One Health approach for in-depth ecosystem health assessments in real-time (33) and in aquaculture to enhance growth, health and overall production sustainability (34). Unfortunately, in spite of being an integral part of the holobiont, the role of parasites in this intricate evolutionary unit has been largely ignored.

Parasites, including helminths, inhabit diverse niches within their hosts, where they inevitably encounter a plethora of commensal, mutualistic and pathogenic microorganisms. These interactions can significantly influence the host immune response, metabolism, overall health and development, both in animal models and humans (12, 13, 35, 36). Despite recent progress (37–39), our understanding of the molecular and cellular mechanisms governing parasite-microbiome dynamics remains limited. Addressing this gap is crucial, as the interplay between parasites and the microbiome might have far-reaching effects on progression of diseases, effectiveness of therapeutic interventions, and development of novel treatments.

Helminths secrete various immunomodulatory molecules that can impact the host immune system directly or alter the composition and function of the (gut) microbiota (14, 40), potentially creating environments more favorable for the parasite while dampening host immune responses. Conversely, certain microbiota may influence parasite colonization and pathogenicity, implying a bidirectional relationship (38). Exploring these parasite-microbiome interactions is essential not only for advancing our understanding of parasitology but also for unlocking new avenues in microbiome research and therapeutic innovation. These may lead to novel microbiome-targeted strategies for managing parasitic infections and novel interventions/treatments that harness the beneficial effects of helminths. Unfortunately, research on the microbiome associated with helminths or other parasites has not kept track with the general surge in microbiome studies (41).

**Figure 1:**
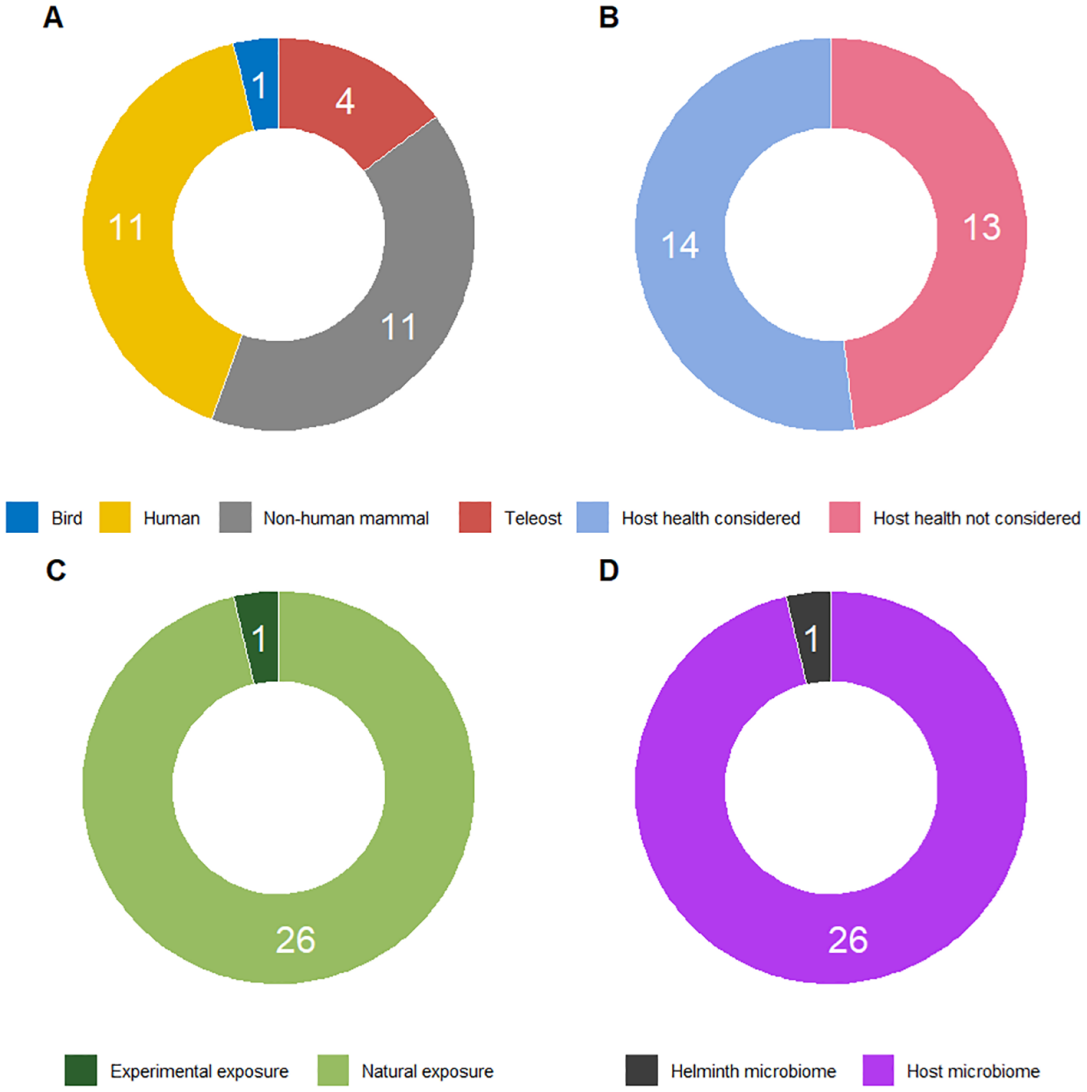
Characterisation of empirical research about the relationship between the diversity of helminths of a host and microbial diversity, based on the following Web of Science search string on 27 August 2024: “diversity AND microb*” (Topic) NOT “Review OR Editorial Material” (Document Type) AND “helminth OR trematod* OR fluke OR cestod* OR tapeworm OR digenean OR monogen* OR nematod* OR acanthocephalan OR worm OR leech OR hirud*” (Topic) AND “health” (Topic). From the resulting 244 hits (**Supplementary Table 1**), 27 were retained, and 179 were not considered either because they did not contain empirical work (they were e.g. a review paper, perspective paper, proceedings paper, database paper, meta-analysis, or methodological paper), or because they did not deal with parasitic worms at all, or only studied helminths in the context of plant parasites, environmental samples, or waste(water). A further 38 were not retained because helminth diversity was not taken into consideration, i.e. these studies either focused on a single species, considered all helminth infections together, or only lumped several species into a single higher-order taxon. For the 27 studies retained, **(A)** shows the distribution over focal host taxa; **(B)** indicates whether studies look at health outcomes for the host, which we considered to be the case when host health was empirically studied, beyond the mere presence or clearance of infection; **(C)** compares whether studies applied experimental infection or relied on natural exposure to helminths; and **(D)** illustrates the proportion of studies focusing on the microbiome of helminths rather than on the microbiome of their hosts.

The importance of microbial community composition and the beneficial role of a diverse microbiome on health outcomes of animals, plants, and ecosystems are well-established (e.g., 42, 43). For instance, they may determine the resilience of a host or ecosystem to environmental perturbations (44, 45). The literature on helminth-microbiota interactions, however, typically focuses on laboratory-kept hosts and single-species infections of helminths (46), overlooking the aspect of parasite diversity at the species and genomic level. To assess the extent to which diversity is taken into account in the study of microbiome-helminth interactions, we performed a search on Web of Science. The search for research on the relationship between host helminth diversity and microbial diversity, resulted in 244 initial hits (**Supplementary Table 1**) and was filtered to 27 relevant studies based on specific criteria such as focus on empirical work, relevance to parasitic worms, and inclusion of helminth diversity, while categorizing these studies by host taxa, health outcomes, and type of helminth exposure (natural or experimental) (**Figure 1**). Twenty-seven publications were retained, which indicates that only a minority of studies on microbiome-parasitic worm interactions includes aspects of helminth (species) diversity. Several studies mentioned the presence of multiple species of helminths or other parasite taxa, while investigating the microbial interactions with a single one (47–49). **Figure 1** details the state of empirical research about the relationship between the diversity of helminths of a host and microbial diversity. Out of the 27 retained studies, 22 investigated human or other mammal hosts (**Figure 1A**). Only just over half of the studies verified health outcomes for the host (**Figure 1B**). Only one publication investigated helminth infections resulting from experimental rather than natural exposure (**Figure 1C**), and a single study focused on the microbiome of helminths rather than of the host itself (**Figure 1D**).

The influence of the host’s environment and behavior on its symbionts makes it hard to mimic a real-life symbiome, or natural exposure to symbionts (50), experimentally. Indeed, the microbiomes of conspecific natural and captive (including laboratory) animal populations are known to differ (e.g., 51, 52), as do the microbial and parasite load between rural and urban populations (53–55). Moreover, in humans and other animals, lifestyle and environment are key factors in determining microbiome composition (56–60). Therefore, approaches going beyond laboratory conditions gained traction in immunology (61) and may be highly relevant to studying the symbiome. For instance, releasing test animals into a semi-natural environment, or oral inoculation, may allow fungal colonization enriching the intestinal microbiota (62). Such approaches are also promising when studying helminth infections and their immune responses: they allow taking into account host and environmental factors that facilitate translation to a natural context. Hence, although experiments are definitely needed to reveal the mechanisms behind helminth-microbiome interactions (63), it remains hard to include the diversity of helminths into experimental approaches. Unsurprisingly, in our literature sample which excluded single-helminth species studies, only a single study applied experimental co-infection: Bonde et al. (64) inoculated pigs with the nematodes *Ascaris suum* and *Oesophagostomum dentatum*. This clearly illustrates that investigations of helminth-microbiota interactions rely on natural host populations when the entire helminth community is to be considered. Unfortunately, such studies are scarce (46) and it is timely to propose additional suitable candidate models to study the health and disease outcomes of helminth-microbiome interactions.

## 3 Candidate models to study helminth-microbiome interactions should be found in the Global South

**Figure 2:**
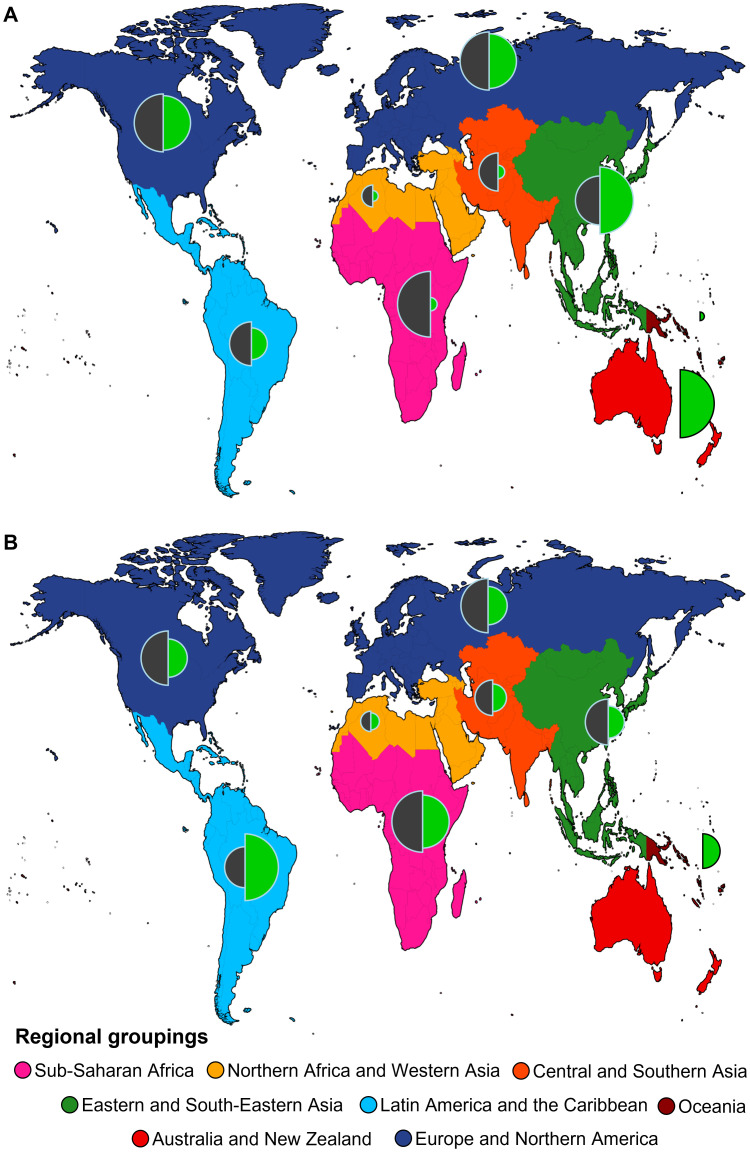
Global distribution of empirical research about the relationship between the diversity of helminths of a host and its microbial diversity (see legend of **Figure 1** for search string) across regional groupings used for indicators and reporting regarding the Sustainable Devevelopment Goals, with colours on the map denoting different regional groupings (https://unstats.un.org/sdgs/indicators/regional-groups/). **(A)** Proportional research effort on helminth and microbial diversity (proportional to the radius of the left half-circles) compared to recent research effort on helminths relative to their known diversity in a given regional grouping (proportional to the radius of the right half-circles). For the latter, we divided the number of available nucleotide sequences on NCBI GenBank per region, by the aggregated total for the region of per-country data of host-helminth records, based on the data and analyses from Poulin et al. (65). This provides an estimate of genetic research effort relative to the regional helminth diversity; in a region where this proportion is low, the helminth diversity has not been adequately studied with recent molecular tools. **(B)** Proportional research effort on helminth and microbial diversity (proportional to the radius of the left half-circles) compared to aggregated per-country prevalence of cysticercosis (proportional to the radius of the right half-circles) (per 100 000 people, based on data from the Global Burden of Disease study, Institute for Health Metrics and Evaluation, taken from https://ourworldindata.org/grapher/prevalence-cysticercosis). We opted to use data on cysticercosis here, as it is a neglected tropical disease that is also transmitted locally in countries of the Global North, and hence not only a public health challenge in the Global South (66, 67).

We mapped the geographical distribution of the helminth-microbiome studies which take into account the diversity of helminths (resulting from the above-mentioned Web of Science search). **Figure 2** shows where the empirical research took place, across the regional groupings in the framework of the Sustainable Development Goals^1^. We compared this with the state of exploration of helminth diversity and the availability of genetic resources (**Figure 2A**) (65), and with the prevalence of cysticercosis (caused by the tapeworm *Taenia solium*), as a proxy for the burden of helminthiases (**Figure 2B**).

While the total number of studies retained in our search is low, they appear quite evenly spread across the globe (**Figure 2**). It is noteworthy that the Global South has seen a comparable amount of research attention in this field. In this region the known and estimated helminth diversity are highest (**Figure 2A**), as is the maximal expected proportion of undiscovered helminth species (68). It is also where the highest cysticercosis prevalences are found (**Figure 2B**). Indeed, although next to cysticercosis there are other helminthiases that occur in the Global North and South (e.g., ascariasis caused by nematode species belonging to *Ascaris*: 69), the highest toll of helminthiases is typically found in the Global South and Africa in particular (70, 71). Therefore, it is fitting that the latter continent is well-covered in certain areas of helminthology, such as geospatial mapping of human helminthiasis (72).

On the other hand, the relative lack of genetic resources in many parts of the Global South, especially sub-Saharan Africa (**Figure 2A**) suggests that its considerable helminth diversity has been insufficiently studied, particularly with regard to modern integrative approaches. This complicates helminth microbiome research and its translation into practice, as it limits, for instance, the possibility to diagnose and identify species and strains by molecular means. Globally, metabarcoding initiatives have done little to mitigate this situation, as they contributed rather to our understanding of occurrences of different microbial (e.g., 73) and helminth communities (reviewed in 74) than patterns of their potential reciprocal interactions. Thus, we commend calls for more parasitological research in the Global South (75); needless to say, this includes capacity development and technology transfer (e.g., 76). In particular, the situation in Africa shows the highest contrast between the available helminth genetic resources and helminth disease burden (**Figure 2**) (see also 77). We believe that work on how helminth diversity patterns influence helminth-microbe interactions therefore also requires more multidisciplinary attention for helminths, including sequencing efforts focusing on host populations under natural exposure.

## 4 Proposal for a fish host model to study helminth-microbiome interactions in real-life settings

Fish models provide effective platforms for understanding the mechanisms and developing therapeutic agents for various human conditions, for instance cancer, cardiovascular, bone, renal and blood diseases (78), and hypercholesterolemia (79). They are also well-established for infectious diseases (80–82), including those directly influenced by microbial communities (83). A key advantage is that fish gut microbiomes can be manipulated to establish the link between host symbiome diversity, health and disease (84). Alterations of the gut microbiota in fish can be achieved directly by fecal material transplant (85) or through indirect approaches, such as diet and dietary inclusion of prebiotics and probiotics (86, 87) as well as antimicrobials (84). Vargas-Albores et al. (88), Luna et al. (89) and Zhang et al. (90) provided overviews of possibilities and knowledge gaps. For example, Rimoldi et al. (91) showed that diets containing insect meal of *Hermetia illucens* significantly increased the abundance of beneficial bacteria in the gut and improved intestinal health in gilthead seabream (*Sparus aurata*) farmed inshore.

Several factors contribute to the establishment of many (tropical) fish species as invaluable laboratory models, namely short generation time (enabling individual monitoring throughout lifespans in experimental settings; in some species even multigenerational studies are possible in the time frame of typical research projects), high fecundity rate and external development, which facilitate genetic and functional studies. Moreover, genome assemblies of many fish species are publicly available^2^. Overall, the gene complements of fishes and tetrapods, including humans, are sufficiently conserved to allow insights into molecular mechanisms obtained in the former to inform our understanding in the latter, including aspects of the immune system. Remarkably, approximately 82% of the genes that have been linked to human diseases have counterparts (i.e., orthologues) in the zebrafish (*Danio rerio*) genome (92). Overall, these features facilitate possibilities of precision genome editing to design human disease models. Tessadori et al. (93) introduced patient alleles in their zebrafish orthologues to model specific cardiovascular disorders, enabling the development of novel therapeutic strategies.

The zebrafish is the most widely recognized fish model of human diseases (78) but not the only one. Teleost fishes, the largest and most diverse group of vertebrates, exhibit an extraordinary range of specialised phenotypes that reflect their adaptation to a wide array of ecological niches (94). Due to its unique life cycle, the turquoise killifish (*Nothobranchius furzeri*), for instance, has emerged as a model for studying ageing and age-related neurological diseases (95). Medaka (*Oryzias latipes*) serves as a model for neurodegenerative conditions like Parkinson’s disease (96). Recently, unusual fish evolutionary mutant models have gained popularity for phenotypes that mimic maladaptive human diseases but are beneficial for the species’ adaptation to its environment (97). Elephantfishes (mormyrids) and knifefishes (gymnotiforms) have experienced numerous natural mutations in the ion channel gene *scn4aa*, making them excellent species for modelling channelopathies affecting skeletal muscle (98). Other examples of fish evolutionary mutant models include the hybrid *Xiphophorus* (*X. maculatus* x *X. helleri*) (99), the mummichog (*Fundulus heteroclitus*) (100) and the Mexican cavefish (*Astyanax mexicanus*) (101), as models of malignant melanoma, mitochondrial, and diabetes and metabolic diseases, respectively.

We propose that fishes, currently only represented by a single study in our sample (**Figure 1A**), are an ideal system to observationally and experimentally study helminth-microbiome interactions and their effect on host health. Fishes are the ecologically dominant vertebrates in many aquatic environments (102). Their immune systems, while simpler than those of mammals, share key components with humans (103). This similarity allows for investigating how parasitic infections and microbiome changes impact the immune system, thereby enhancing our general understanding of how such interactions influence health outcomes.

Given the need for natural candidate models and helminthological research in the Global South in general and in Africa in particular (**Figure 2**), and the above-mentioned potential of fishes, we propose that a well-delineated aquatic system in the tropics, such as the African Great Lakes, could provide an ideal system to investigate helminth-microbiome interactions under natural conditions. Disease burden of tropical water-borne diseases is societally relevant in the region (75) and contact with water as homogenizer exposes the human population to the same naturally occurring parasites as other potential hosts in the ecosystem. The increasing availability of high-quality reference fish genomes facilitates the application of various -omics approaches, especially for cichlid fishes (104–107), the most species-rich fish taxon in these lakes (108). Cichlids, for which also an elaborate experimental toolbox is available (109), have been proposed as models for both non-communicable (110) and infectious diseases, with the African Great Lakes cichlids among the best-studied ones in terms of metazoan parasitological baseline (111). Also, other African cichlid species, especially economically important ones such as Nile tilapia and other tilapias, are well-studied parasitologically (112, 113), though health aspects are rarely considered (114). Elsewhere in the Global South, several Neotropical cichlids are well-studied (109) also in terms of their parasites (115–120). Moreover, also the microbial symbionts of (African Great Lake) cichlids have recently seen a surge in scientific attention (121, 122). In turn, cichlids sourced from such diverse natural study systems, may be well-suited to develop as model organisms for experimentally manipulating both microbiomes and parasitic load under lab conditions, enabling researchers to uncover causal relationships and test potential therapeutic interventions relevant to human health.

## Data Availability

The original contributions presented in the study are included in the article/**Supplementary Material**. Further inquiries can be directed to the corresponding authors.

## Footnotes

1 https://unstats.un.org/sdgs/indicators/regional-groups/

2 https://www.ensembl.org
